# Tomato SlWRKY3 Negatively Regulates *Botrytis cinerea* Resistance via *TPK1b*

**DOI:** 10.3390/plants13121597

**Published:** 2024-06-08

**Authors:** Dan Luo, Jun Cai, Wenhui Sun, Qihong Yang, Guoyu Hu, Taotao Wang

**Affiliations:** 1 College of Horticulture, Shanxi Agricultural University, Jinzhong 030801, China; 2 National Key Laboratory for Germplasm Innovation and Utilization of Horticultural Crops, Huazhong Agriculture University, Wuhan 430070, China; 3 Guangxi Academy of Agricultural Science, Nanning 530007, China

**Keywords:** tomato, *Botrytis cinerea*, *SlWRKY3*, *TPK1b*, resistance

## Abstract

*Botrytis cinerea* is considered the second most important fungal plant pathogen, and can cause serious disease, especially on tomato. The *TPK1b* gene encodes a receptor-like kinase that can positively regulate plant resistance to *B. cinerea*. Here, we identified a tomato WRKY transcription factor SlWRKY3 that binds to the W-box on the *TPK1b* promoter. It can negatively regulate *TPK1b* transcription, then regulate downstream signaling pathways, and ultimately negatively regulate tomato resistance to *B. cinerea. SlWRKY3* interference can enhance resistance to *B. cinerea*, and *SlWRKY3* overexpression leads to susceptibility to *B. cinerea*. Additionally, we found that *B. cinerea* can significantly, and rapidly, induce the upregulation of *SlWRKY3* expression. In *SlWRKY3* transgenic plants, the *TPK1b* expression level was negatively correlated with *SlWRKY3* expression. Compared with the control, the expression of the SA pathway marker gene *PR1* was downregulated in *W3-OE* plants and upregulated in *W3-Ri* plants when inoculated with *B. cinerea* for 48 h. Moreover, *SlWRKY3* positively regulated ROS production. Overall, *SlWRKY3* can inhibit *TPK1b* transcription in tomato, and negatively regulate resistance to *B. cinerea* by modulating the downstream SA and ROS pathways.

## 1. Introduction

*Botrytis cinerea* is a necrotrophic pathogen, which invades through the stomata, wounds, and other plant parts; triggers enzymolysis and releases phytotoxins; decomposes and eliminates host cells; and self-replicates and reproduces after successfully entering host cells. *B. cinerea* can invade the stems, leaves, flowers, fruits, and other organs of more than 200 plant species, seriously threatening global fruit and vegetable yield and quality. In production, chemicals are the main control method, but they not only affect food quality, but also potentially harm the environment. Therefore, it is particularly important to determine the *B. cinerea* resistance mechanism and cultivate resistant varieties. *B. cinerea* infects plants and produces an immune response in a process of mutual competition and coevolution [1].

Plants experience biological stress caused by pathogen infection, but can recognize stress-generated signals and trigger their natural immune system, resulting in a defense response [1]. The plant innate immunity concept originated from animal studies, and was systematically outlined in 2002 [2]. In short, PTI (PAMP-triggered immunity) and ETI (Effector-triggered immunity) coexist in plants to respond to pathogen infection. The immune signals produced by a pathogen infection can be transmitted by complex biomolecular networks in plants, thus regulating immune response at all levels. For example, MAPK cascades (mitogen-activated protein kinases) are indispensable components of plant immune signal transduction [3]. Transcription factors such as WRKY, bZIPs, and MYB can act as target MARK proteins, regulate downstream gene expression, and transmit immune signals [4]. Hormone signal transduction is the most extensive component of plant immune signal transduction, and changes in auxin (IAA), ethylene (ET), salicylic acid (SA), and jasmonic acid (JA) hormone levels can play a direct role in immune response [5,6,7,8]. Strigolactones (SLs) are a class of relatively new plant hormones that play a pivotal role in maintaining the structural integrity of plants. They are instrumental in regulating lateral branching and modulating the plant’s response to a variety of abiotic stresses [9]. Pathogenic fungus triggers the defense response of plants. In response, the plant initiates intricate networks of signaling pathways, culminating in the induction of hypersensitive responses (HRs) and/or the acquisition of systemic acquired resistance (SAR). The HR response is accompanied by the activation of programmed cell death (PCD), which is typically characterized by oxidative burst. This burst results in the accumulation of reactive oxygen species (ROS) such as superoxide anions, hydrogen peroxide, and hydroxyl radicals. These reactive oxygen species serve as signaling molecules, produced by a variety of enzymatic and non-enzymatic processes, and are integral to the defense mechanisms that are triggered during the plant–pathogen interaction. While low levels of ROS act as pivotal signal transducers, excessive ROS can exert a potent oxidative force, leading to detrimental effects on the plant [10].

Receptor kinases (RKs) and receptor-like protein kinases (RLKs) recognize various pathogens early and play a key role in defense and symbiosis. Plant RLKs are a kind of plant protein kinase, which can transfer phosphate groups from donors to substrate protein receptors, realize substrate protein trans-phosphorylation, and complete protein activity regulation and signal amplification and transmission [11]. Plant cytoplasmic receptor-like kinases (RLCK) are an RLK subfamily, and can be widely involved in the transduction of related signals such as defense, growth and development, and hormones [12]. BIK1 is a typical RLCK in *Arabidopsis thaliana* that can interact with complexes formed by two RLKS, FLS2 and BAK1, and participate in necrotic pathogen-induced immune and ethylene-mediated defense responses [13]. It can also participate in Ca^2+^ signal transduction, reactive oxygen species production, and other immune pathways [14,15]. Additionally, it can also participate in the regulation of hormones such as ET, JA, and SA [14,16,17]. The tomato *TPK1b* gene encodes a functional cytoplasmic receptor-like kinase with phosphorylation activity, which is independent of JA synthesis and response, and mediates the regulation of plant defense response to necrotic fungi and herbivorous insects through ET [18]. *TPK1b-RNAi* transgenic lines reduced resistance to *B. cinerea* and insects, confirming that they play an important role in tomato defense response. Moreover, *TPK1b* in tomato and *BIK1* in *Arabidopsis* may perform similar biological functions [18,19]. Additionally, the cerato-platanin family protein BcSpl1 and glycoprotein BcGs1 isolated from *B. cinerea* can upregulate *TPK1b* expression in plants [20,21]. However, the *TPK1b* regulation mechanism in immune response is still unclear, and deserves further study.

Plant transcription factors can regulate the expression of stress-related genes at the transcription level and then regulate the plant immune response. The WRKY transcription factor is a plant-specific transcription factor, and belongs to one of the largest transcription factor families in plants. Numerous WRKY transcription factors occur in >20 plants, including rice, soybean, and *Arabidopsis thaliana* [22], and 81 WRKY transcription factors have been identified in tomato [23]. The names of the WRKY transcription factors reflect that this protein family contains a conserved domain consisting of 60 amino acids, a domain N-terminal that contains seven absolutely conserved amino acids with the WRKYGQK sequence, and a C-terminal with a special zinc finger structure [24]. Based on the number of domains and zinc finger structure characteristics, WRKY transcription factors can be divided into categories I, II, and III [25]. WRKY transcription factors can bind to the specific cis-acting element W-box (TTGACC/T) in its target gene promoter, thus regulating target gene transcription. WRKY transcription factors play an important role in plant immune response. In pepper, *CaWRKY1* silencing leads to a decrease in *Xanthomonas* leaf growth, while HR response caused by tobacco mosaic virus and *Pseudomonas* in *CaWRKY1* overexpression plants is more rapid [26]. The mutant *wrky27-1* in *Arabidopsis* displayed a phenotype that delayed the development of bacterial wilt disease [27]. *WRKY11* and *WRKY17* could negatively regulate plant resistance to *Pseudomonas syringae* [28], while *WRKY3* and *WRKY4* could regulate *Arabidopsis* resistance to necrotic pathogens, and *WRKY4* could also regulate plant resistance to biotrophic pathogens [29]. Additionally, *OsWRKY53* can control the scale of early defense responses [30]. *HvWRKY1* plays an important role in the resistance of barley to leaf stripe disease [31]. Tobacco *WRKY8* can regulate plant sensitivity to pathogens such as *Phytophthora infestans*, and *SlWRKY1* and *SlWRKY33* can regulate plant resistance to *B. cinerea* [32,33,34]. *SlWRKY3* is an important regulator of tomato salt tolerance [35] and can be used as a positive regulator of the tomato–*Phytophthora* interaction, enhancing tomato resistance to *Phytophthora* [36].

Previous research has identified TPK1b as a positive regulator of tomato resistance against *B. cinerea* [18]. In our current investigation, we have ascertained that SlWRKY3 can bind to the W-box element on the *TPK1b* promoter, as evidenced by our screening of a yeast one-hybrid library and subsequent experimental validation. While *TPK1b’s* role in tomato resistance is well documented, the specific contribution of *SlWRKY3* to the defense mechanisms against *B. cinerea* has been less explored. To elucidate the potential involvement of *SlWRKY3* in modulating tomato resistance to *B. cinerea* via *TPK1b*, we conducted inoculation experiments with *SlWRKY3* transgenic plants. Our results revealed a surprising negative regulatory role of *SlWRKY3* in tomato resistance to *B. cinerea*. Furthermore, we observed that *TPK1b* expression was diminished in *SlWRKY3* overexpression (*OE*) plants and increased in *SlWRKY3* RNA interference (*Ri*) plants. The outcomes of this study suggest that *SlWRKY3* exerts a negative regulatory effect on tomato resistance to *B. cinerea* by directly suppressing the transcriptional activity of *TPK1b*. This revelation not only deepens our comprehension of the intricate molecular mechanisms at play in tomato resistance but also offers novel insights that could inform the development of strategies aimed at bolstering crop resistance to *B. cinerea*.

## 2. Results

### 2.1. Analysis of the TPK1b Gene Promoter and Screening Library for Yeast One Hybridization

**Figure 1:**
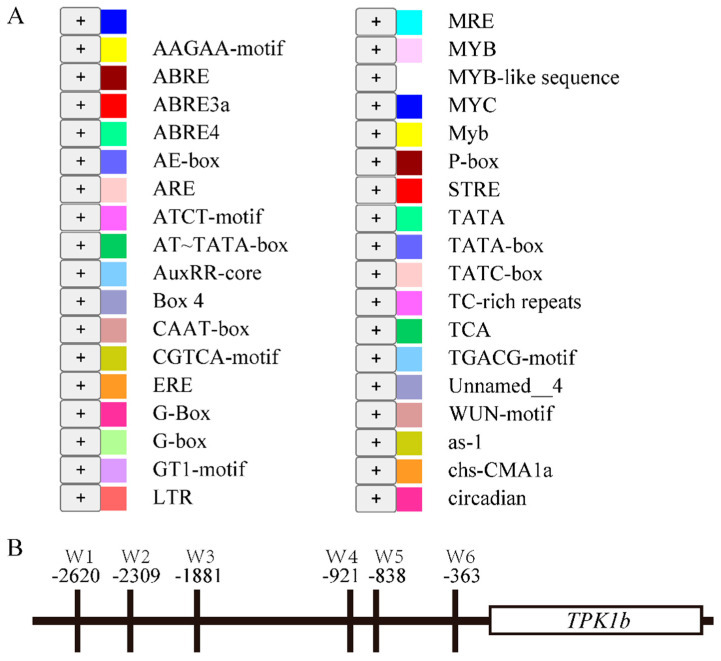
Cis-acting elements on the *TPK1b* promoter: (**A**) Prediction of cis-acting elements on the *TPK1b* promoter based on the PlantCARE database. (**B**) Distribution of W-box elements in the *TPK1b* promoter.

To find the upstream regulatory factors, we selected a *TPK1b* promoter fragment (−1 to −3000 bp) and predicted the cis-acting elements on it using the PlantCARE database (https://bioinformatics.psb.ugent.be/webtools/plantcare/html/ (accessed on 8 May 2019)). They were mainly related to light response, followed by hormone-related elements such as abscisic acid, auxin, and JA, and also included some elements related to other life activities such as anaerobic induction, defense response, and circadian rhythm regulation (Figure 1). Simultaneously, we found six W-box elements that can be combined by WRKY transcription factors (Figure 1B). Subsequently, the yeast one-hybrid screening system was used to screen the *TPK1b* promoter fragment (−1 to −3000 bp), and five transcription factors were identified (Table 1), including two bHLH transcription factors, two ERF transcription factors, and one WRKY transcription factor.

### 2.2. SlWRKY3 Negatively Regulates Tomato Resistance to B. cinerea

**Figure 2:**
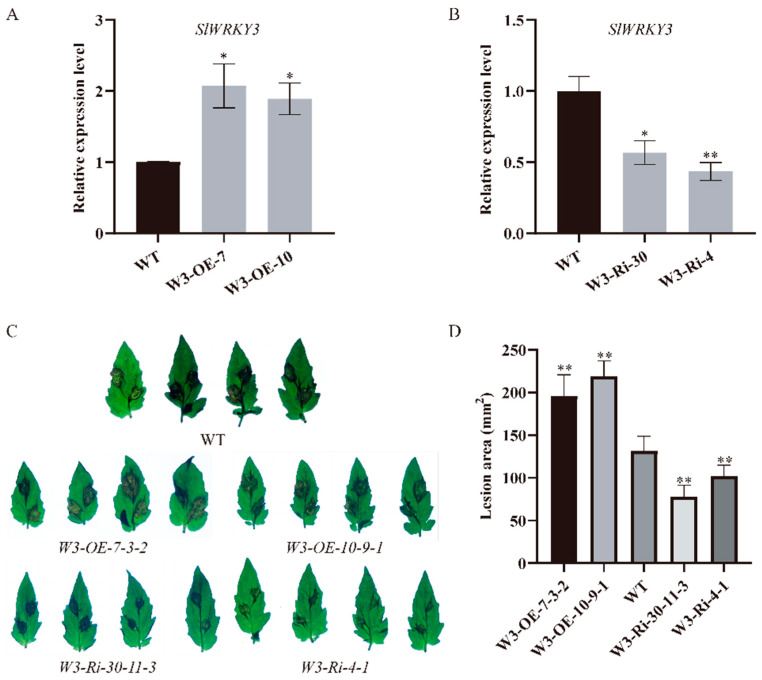
*SlWRKY3* negatively regulates tomato resistance to *B. cinerea*: (**A**,**B**): *SlWRKY3* transgenic line *SlWRKY3* expression level detection, (**A**): *SlWRKY3* overexpression lines, (**B**): *SlWRKY3* interference lines; (**C**): phenotype of *SlWRKY3* transgenic lines inoculated with *B. cinerea*; (**D**): the lesion area of *SlWRKY3* transgenic line inoculated with *B. cinerea*. (Dunnett-*t* test *p* * < 0.05, *p* ** < 0.01.)

To explore whether the five transcription factors obtained from the *TPK1b* promoter yeast one-hybrid screening experiment are involved in tomato resistance to *B. cinerea*, we conducted tomato genetic transformation on these five transcription factors. However, only *SlWRKY3* transgenic plants had significant changes in resistance to *B. cinerea*. Therefore, we focused on the effect of *SlWRKY3* on resistance to *B. cinerea* and its relationship with *TPK1b*. Firstly, we detected the relative expression level of *SlWRKY3* transgenic plants, and found that it increased significantly in overexpression lines (*W3-OE*), while it decreased significantly in interference lines (*W3-Ri*) (Figure 2A,B). Through inoculating detached leaves with *B. cinerea* spores, we found that *W3-OE* plant lesion areas were significantly larger, while *W3-Ri* plant lesion areas were significantly smaller than the WT (Figure 2C,D). The results showed that *W3-OE* plant resistance to *B. cinerea* decreased compared with the control, while *W3-Ri* plants had higher *B. cinerea* resistance. Moreover, they indicate that *SlWRKY3* could negatively regulate tomato plant resistance to *B. cinerea*.

### 2.3. SlWRKY3 Is an Evolutionarily Conserved Type I WRKY Transcription Factor Highly Expressed in Flowers and Leaves

**Figure 3:**
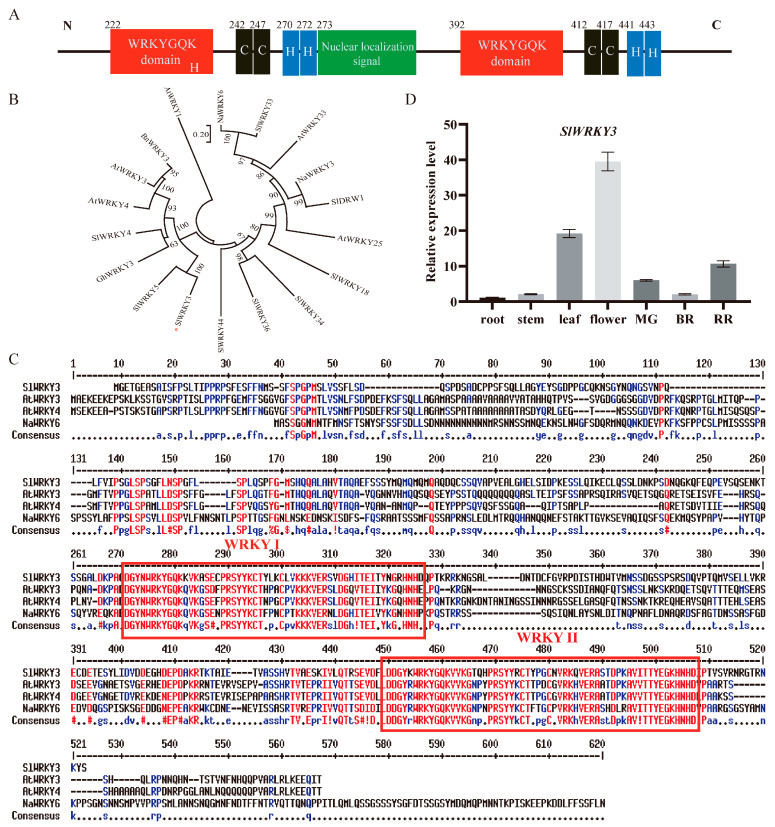
*SlWRKY3* sequence analysis and tissue expression: (**A**): *SlWRKY3* sequence structure; (**B**): phylogenetic tree of some type I WRKY transcription factors (Sl: tomato; Bn: Rape; Na: wild tobacco; At: *Arabidopsis thaliana*; GH: upland cotton); (**C**): amino acid sequence alignment of several type I WRKY transcription factors; (**D**): *SlWRKY3* tissue expression profile. MG: green ripening stage; BR: discoloration period; RR: red ripening stage.

WRKY transcription factors can be classified into three categories according to the number of WRKY functional domains and the zinc finger motif type. By analyzing the *SlWRKY3* amino acid sequence, we found that it contains two typical WRKY functional domains with WRKYGQK sequences, and two C2H2 zinc finger motifs (Figure 3A), which belong to type I WRKY transcription factors. Furthermore, there is also a nuclear localization signal between the two WRKY functional domains. We compared it with the other three type I WRKY transcription factors and found that they have high sequence homology (Figure 3B). According to the phylogenetic tree analysis, *SlWRKY3* is closely related to type I WRKY transcription factors in tomato and other species, indicating that its evolutionary relationship is conservative (Figure 3C). *AtWRKY3* and *AtWRKY4*, which are closely related to *SlWRKY3*, have been reported to regulate plant defense response to biological stress. *SlWRKY3* expression in tomato roots, stems, leaves, and flowers, and young, green-ripe, and red-ripe fruits was detected. Furthermore, *SlWRKY3*’s relative expression was very low in roots and stems, but high in flowers, leaves, and red-ripe fruits (Figure 3D).

### 2.4. SlWRKY3 Can Bind to Two W-Boxes on TPK1b Promoter

**Figure 4:**
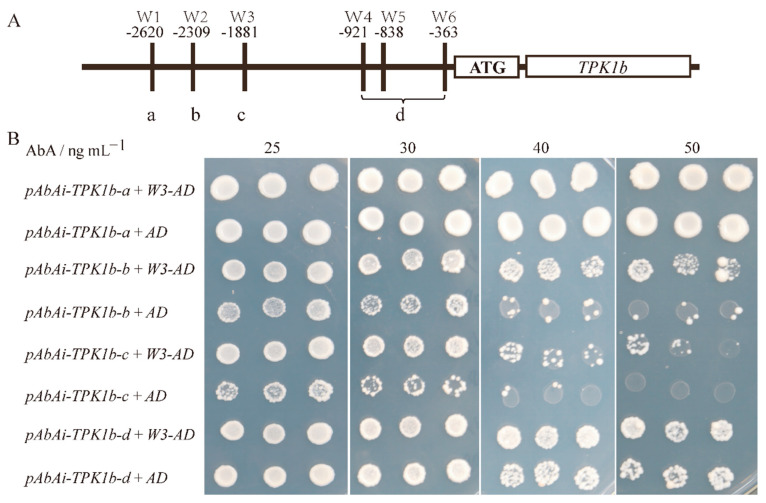
SlWRKY3 and *TPK1b* promoter yeast one-hybrid experiment: (**A**): *TPK1b* promoter segmentation based on W-box position; (**B**): SlWRKY3 and *TPK1b* promoter yeast one hybridization. The yeast strain Y1HGold was transformed with the bait vector *pAbAi-TPK1b* and the prey vector *SlWRKY3-AD* (*W3-AD*) and plated on SD/-Leu-Ura medium containing different concentrations of aureobasidin A (AbA). The empty *pGADT7* vector (*AD*) was used as a negative control.

The yeast one-hybrid assay indicated that SlWRKY3 could interact with the *TPK1b* promoter, but the SlWRKY3 binding site on the *TPK1b* promoter was unclear. It has been reported that the WRKY transcription factor regulates target gene transcription expression by binding with W-box, a cis-acting element on the target gene promoter. To determine the binding site, we found six W-boxes on the *TPK1b* promoter (−1 to −3000 bp)-positive and -negative DNA strands. Based on the location of these six W-boxes, we divided the promoter into four fragments, a, b, c, and d (Figure 4A, where a, b, and c each contain one W-box, and d contains three W-boxes), and carried out yeast one-hybrid experiments with *SlWRKY3,* respectively. On SD/-Ura-Leu of 50 ng·mL^−1^ aureobasidin A (AbA), we observed that the *TPK1b-b* and *TPK1b-c* groups’ yeast growth rate was significantly faster than blank control *pGADT7*, while there was no significant difference between the *TPK1b-a* and *TPK1b-d* promoter groups and the control. The results indicate that *SlWRKY3* could bind to the W-box *TPK1b* promoter’s b and c parts, and then regulate *TPK1b* transcription.

### 2.5. SlWRKY3 and TPK1b Expression Was Negatively Correlated and SlWRKY3 Could Be Significantly Induced by B. cinerea

**Figure 5:**
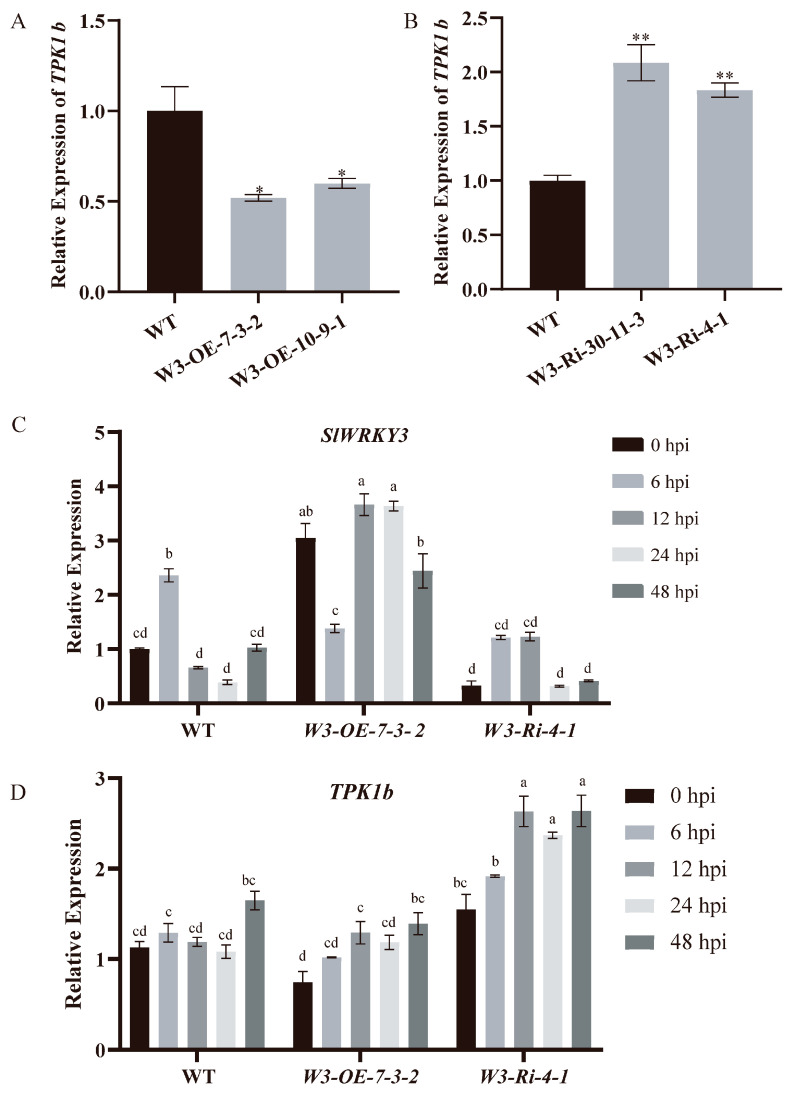
*SlWRKY3* and *TPK1b* expression levels: (**A**,**B**): Relationship between expression levels of *SlWRKY3* and *TPK1b* in *SlWRKY3* transgenic lines. (Dunnett-*t* test *p* * < 0.05, *p* ** < 0.01.) (**C**,**D**): Changes in expression in *SlWRKY3* and *TPK1b* in *SlWRKY3* transgenic lines inoculated with *B. cinerea*. The relative gene expression levels at 0, 6, 12, 24, and 48 h post inoculation (hpi) of *B. cinerea* were detected by real-time fluorescent quantitative PCR technology. Statistically significant differences were determined using a One-Way ANOVA. Different letters indicate statistically significant differences.

To clarify the regulatory relationship between *SlWRKY3* and *TPK1b*, their relative expression levels in the leaves of *SlWRKY3* transgenic lines were detected by RT-qPCR. *TPK1b* expression levels in *W3-OE* lines decreased significantly compared with WT, while in *W3-Ri* lines they increased significantly (Figure 5A,B), indicating that the *TPK1b* expression level was regulated by *SlWRKY3* in tomato plants, and negatively correlated with it.

Both *SlWRKY3* and *TPK1b* can change tomato resistance to *B. cinerea*, so we investigated whether their expression relationship changed due to *B. cinerea* infection. *W3-OE*, *W3-Ri*, and WT of the same age and size were sprayed with *B. cinerea* spores, and *SlWRKY3* and *TPK1b* expression levels were detected in their leaves at 0, 6, 12, 24, and 48 h after inoculation (Figure 5C,D). In the WT and the *W3-Ri* line, the relative expression of *SlWRKY3* exhibited a rapid increase at 6 h post inoculation with *B. cinerea*. In contrast, the relative expression of *SlWRKY3* was observed to be repressed at 6 h post inoculation, followed by a significant increase in the *W3-OE* line. This pattern suggests that *SlWRKY3* acts as an early-response gene to *B. cinerea*. Furthermore, *TPK1b* showed a pronounced induction of expression in both the *W3-OE* and *W3-Ri* lines at 12 h post inoculation with *B. cinerea*. Notably, the relative expression of *TPK1b* in the *W3-Ri* line was generally higher than that observed in the *W3-OE* line when inoculated with *B. cinerea*. *TPK1b* expression was induced at 48 h post inoculation with *B. cinerea* in the WT.

### 2.6. SlWRKY3 Can Regulate Downstream SA and ROS Signaling Pathways

**Figure 6:**
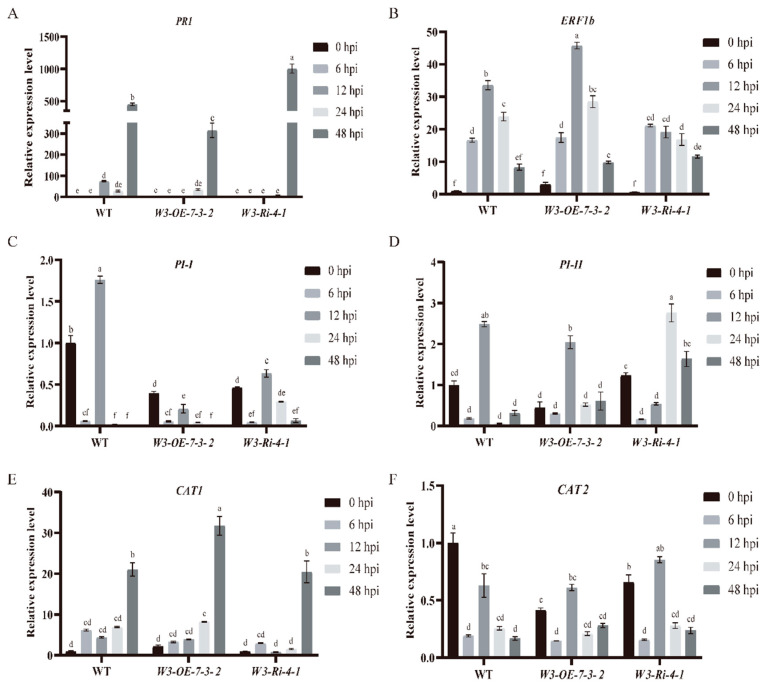
Expression level detection of related genes in SlWRKY3 transgenic plants after *B. cinerea* inoculation. (**A**–**F**): PR1, ERF1b, PI-I, PI-II, CAT1, CAT2 expression levels. Different letters indicate significant differences in gene expression between different time points. The relative gene expression levels at 0, 6, 12, 24, and 48 h post inoculation (hpi) of *B. cinerea* were detected by real-time fluorescent quantitative PCR technology. Statistically significant differences were determined using a One-Way ANOVA. Different letters indicate statistically significant differences.

To investigate which signal pathways are involved in *TPK1b* regulation by *SlWRKY3* to change resistance to *B. cinerea*, we detected the expression levels of some signal pathway marker genes in *W3-OE* and *W3-Ri* plants at 0, 6, 12, 24, and 48 h after *B. cinerea* infection. These included hormone signaling pathways such as *PI-I* and *PI-II* of the JA signaling pathway, *PR1* of the salicylic acid signaling pathway, *ERF1b* of the ethylene signaling pathway, and *CAT1* and *CAT2* of the reactive oxygen species (ROS) signaling pathway. *PR1*’s relative expression levels in the *W3-OE* line at 48 h after *B. cinerea* inoculation were significantly lower than in WT, while in the *W3-Ri* line they were significantly higher (Figure 6A). There was no obvious change in the expression levels of marker genes in the JA and ethylene signaling pathwayS (Figure 6B–D). In the ROS signaling pathway, *CAT1*’s relative expression levels in *W3-OE* lines were significantly higher than in *W3-Ri* lines and WT at 48 h after *B. cinerea* inoculation (Figure 6E). When the plants were not inoculated with *B. cinerea*, the relative expression level of the *CAT2* gene in *SlWRKY3* transgenic plants was significantly lower than that in WT. After inoculation with *B. cinerea*, there was no significant difference in *CAT2*’s relative expression level between *SlWRKY3* transgenic plants and WT (Figure 6F). These results suggest that *SlWRKY3* may regulate resistance to *B. cinerea* by regulating downstream SA and ROS signaling pathways, but whether *SlWRKY3* regulates other pathways remains to be verified.

### 2.7. SlWRKY3 Regulates ROS Production

**Figure 7:**
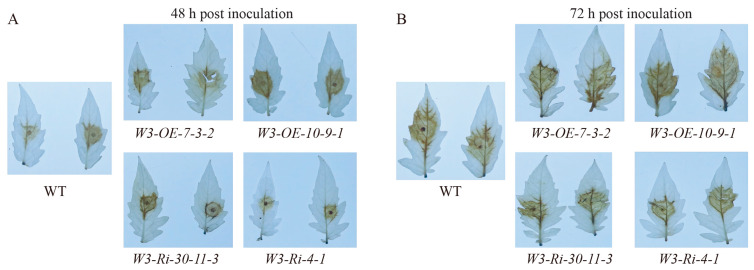
Visualization of ROS production in *SlWRKY3* transgenic plants after *B. cinerea* inoculation. The leaves of *SlWRKY3* transgenic plants were inoculated with *B. cinerea*, and DAB staining was performed at 48 h (**A**) and 72 h (**B**) post inoculation.

The immune response caused by pathogen infection can cause a ROS outbreak in plants, and then trigger a series of immune responses. However, ROS can promote infection by necrotic pathogens. To determine the effect of *SlWRKY3* on ROS production, we used DAB staining to detect ROS production in WT, *W3-OE* and *W3-Ri* line leaves at 0, 12, 24, 48 and 72 h after *B. cinerea* infection. ROS produced in *W3-OE* plants was significantly higher than in WT at 48 and 72 h after being infected by *B. cinerea*, while in *W3-Ri* plants ROS was significantly lower (Figure 7). This indicates that *SlWRKY3* could positively regulate ROS production after *B. cinerea* infection, and we speculate that this aggravates the hypersensitivity reaction caused by ROS, leading to more cell death, which is more conducive to further *B. cinerea* infection.

## 3. Discussion

*B. cinerea* seriously threatens tomato growth, resulting in reduced or even no tomato harvest, and presently, there is a lack of *B. cinerea*-resistant tomato varieties. Although many studies have investigated tomato resistance to *B. cinerea*, little is known about its molecular mechanism. Therefore, it is crucial to further analyze the tomato resistance mechanism against *B. cinerea* in breeding and production. In 2008, the tomato *TPK1b* protein, a cytoplasmic receptor-like protein kinase localized in the plasma membrane, was induced by *B. cinerea* or other bacteria and stresses and shown to positively regulate tomato resistance to *B. cinerea* and insect infestation. Upstream of *TPK1b*, a tomato receptor kinase *PORK1* can directly phosphorylate *TPK1b* and regulate its role in immune response and signal transduction [18,37]. To further explore the molecular mechanism, we cloned the *TPK1b* promoter and conducted a yeast one hybridization screening library experiment. We found that many transcription factors can bind to the *TPK1b* promoter, but not all of them can participate in the immune signal transduction pathway mediated by *TPK1b*. *SlWRKY3* plays a regulatory role in *TPK1b*-mediated immune response, and it solely regulates tomato resistance to *B. cinerea*. According to the WRKY protein classification rules, the SlWRKY3 protein is an evolutionarily conserved type I WRKY transcription factor. Further studies have found that SlWRKY3 can bind to the *TPK1b* promoter through two W-boxes and have a regulatory effect on *TPK1b* transcription.

Certain plant WRKY proteins are known to be integral to the immune response in plants. The WRKY transcription factor RhWRKY30 can promote lignin biosynthesis, improve the resistance of rose petals to *B. cinerea*, and regulate the expression of *RhCAD1* [38]. Similarly, RNA interference (RNAi) targeting *FaWRKY29* and *FaWRKY64* has been demonstrated to enhance the resistance of strawberries to *B. cinerea*. This is achieved by modulating ABA and JA signals, adjusting the composition of the plant cell wall, and regulating the expression of genes associated with ROS [39]. Furthermore, the overexpression of *SlWRKY46* has been observed to suppress the expression of SA and JA marker genes, including *PR1* and protease inhibitors (*PI-I* and *PI-II*), thereby negatively impacting tomato resistance to *B. cinerea* [40]. Additionally, SlWRKY3 has been identified as a positive regulator of tomato defense responses to nematode invasion and salt stress [35,41]. Herein, we discovered that the WRKY transcription factor SlWRKY3 is capable of binding to the *TPK1b* promoter, thereby regulating the immune response mediated by *TPK1b* and ultimately exerting a negative regulatory effect on tomato plant resistance to *B. cinerea*.

Salicylic acid (SA) is a plant hormone that plays an important role in plant immune response, and, more specifically, can mediate the innate antiviral immune response in plants [42]. SA has a close relationship with systemic acquired resistance (SAR) and pathogenesis-related proteins (PRs). SAR reactions are generally thought to be caused by increased PR protein expression [43]. Exogenous SA can stimulate the transfer of PR proteins to generate an SAR immune response. High SA accumulation in plants can enhance pathogen resistance and increase PR protein expression [44]. Conversely, when SA accumulation is blocked, the plant cannot normally produce the ETI or SAR immune response [45]. In plants, multiple WRKY transcription factor family members can participate in SA biosynthesis and SA-mediated immune responses [46]. *PR1* is accumulated downstream of SA and is a marker gene in SA-mediated SAR response. Moreover, the WRKY transcription factor can regulate *PR1* gene expression [47]. We found a significant difference in *PR1* gene expression between *SlWRKY3* overexpression and interference lines after *B. cinerea* inoculation. *PR1* expression level in *W3-OE* lines decreased, while in *W3-Ri* lines it significantly increased. Therefore, we speculate that *SlWRKY3* could participate in the SA hormone signaling pathway through *PR1* expression regulation, and participate in the SAR immune response mediated by SA, thus achieving a negative regulatory effect on tomato’s defense against *B. cinerea* infection. However, since binding of SlWRKY3 and *PR1* promoters has not been verified, it is still unclear whether SlWRKY3’s regulation of *PR1* expression is a direct relationship.

Oxidation, as the second messenger, transmits immune signals and causes hypersensitivity reactions leading to cell death, thus resisting the invasion of some pathogens. However, ROS will weaken plant immunity to necrotic pathogens because cell death will accelerate their invasion. As a typical necrotic pathogen, *B. cinerea* has certain resistance to ROS outbreak, can promote its outbreak in host plants, and produce ROS by itself [48]. ROS in plant disease interactions mainly include superoxide anion O_2_-, hydroxyl free group ·OH, and hydrogen peroxide H_2_O_2_. CAT, as a catalase in plants, can catalyze the H_2_O_2_ decomposition generated by ROS outbreak. Elimination of excess H_2_O_2_ can reduce the plant cell damage and HR response caused by ROS [49]. We detected expression levels of the CAT marker genes *CAT1* and *CAT2* in the *SlWRKY3* transgenic line after inoculation with *B. cinerea*, and found that the *CAT1* expression level in *SlWRKY3-OE* was significantly higher than *SlWRKY3-Ri*. However, there was no significant difference in the relative expression level of *CAT2* between *SlWRKY3* transgenic plants and WT after *B.cinerea* inoculation, so it was impossible to determine how *SlWRKY3* regulates ROS production after *B. cinerea* inoculation. DAB staining was performed on the ROS produced in *SlWRKY3* transgenic plants after *B. cinerea* inoculation, and the ROS produced in *SlWRKY3-OE* after *B. cinerea* infection was significantly higher than in *SlWRKY3-Ri*. These results indicate that *SlWRKY3* can positively regulate ROS production, thereby promoting an ROS-mediated hypersensitivity response in plant cells, which may also explain why *SlWRKY3* negatively regulates tomato resistance to *B. cinerea*. The *CAT1* expression level in *W3-OE* lines was higher than *W3-Ri* lines after *B. cinerea* inoculation. However, it is possible that there are other factors contributing to the increased expression of *CAT1* in *W3-OE* lines, which may not necessarily lead to a reduction in ROS production.

Since the yeast interaction verified the binding of SlWRKY3 and *TPK1b* promoter, their regulatory relationship is a more concerning issue in this study. Based on the mode of tomato resistance to *B. cinerea*, we speculate that *SlWRKY3* could negatively regulate *TPK1b* expression and transcription. However, there are other molecular substances upstream of *TPK1b* such as *PORK1* that can regulate *TPK1b* expression [37]. *SlWRKY3* may not only regulate *TPK1b* transcription through direct binding with the *TPK1b* promoter but also achieve indirect regulatory effects on *TPK1b* through interacting with other factors upstream of *TPK1b* or interacting with *TPK1b* itself. Overall, *TPK1b* expression was negatively correlated with *SlWRKY3* in *SlWRKY3* transgenic plants, but the regulatory mechanism of *SlWRKY3* on *TPK1b* needs further study.

*TPK1b* has been reported to mediate the immune response of tomatoes to necrotic fungi through the ET pathway as a signaling component. We showed that the transcription factor SlWRKY3 not only acts upstream of *TPK1b* but also negatively regulates the immune response mediated by *TPK1b*. It can also regulate immune pathways such as SA and ROS in plants, thereby constructing an immune signal transduction network mediated by *SlWRKY3* that negatively regulates tomato resistance to *B. cinerea*.

## 4. Materials and methods

### 4.1. Plant Materials

Tomato (*Solanum lycopersicum* L.) plants used in this experiment were planted in a greenhouse at 25 °C, 16 h/8 h day and night cycle, and 55% relative humidity. Using the tomato cultivar ‘Alisa Craig’ as the background material, we successfully achieved genetic transformation to obtain the *SlWRKY3* transgenic material. *SlWRKY3* transgenic materials were *SlWRKY3* overexpression transgenic lines *W3-OE-7-3-2* and *W3-OE-10-9-1* and *SlWRKY3* RNA interference transgenic lines *W3-Ri-19-1-2* and *W3-Ri-4-1*.

### 4.2. Genetic Transformation

We successfully integrated the complete coding sequence of *SlWRKY3* into the overexpression vector *pHellsgate8*, resulting in the construction of the *SlWRKY3* overexpression vector (designated as *W3-OE*). Additionally, a 243-base-pair-specific fragment of *SlWRKY3* was precisely inserted into the plasmid *pHellsgate2*, yielding the *SlWRKY3* RNA interference vector (designated as *W3-Ri*). The primer sequences for constructing overexpression and RNA interference vectors are listed in Supplementary Table S1. *Agrobacterium tumefaciens*-mediated transformation was performed to generate *SlWRKY3* transgenic plants [50]. Tomato cultivar ‘Alisa Craig’ (LA2838A) was used as the wild-type background. Tomato seeds were soaked in distilled water for 30 min and then decanted. Then, 75% alcohol was added, shaken for 30~60 s, and then decanted, and 2% sodium hypochlorite solution was added and shaken for 15 min, and then the seeds washed with sterile water 3 times. Cleaned seeds were inoculated on 1/2 MS medium and cultured for 6~8 days until two cotyledons had fully unfolded. Explants with 2–3 segments of cotyledons were cultured in the dark on KCMS medium for 1 day. The activated *Agrobacterium* line was cultured overnight, centrifuged at 3500 r·min^−1^ for 5 min, and then the supernatant was removed and suspended in 0.2 MS liquid medium. Pre-cultured cotyledons were transferred to 0.2 MS liquid medium for re-suspension. An appropriate amount of *Agrobacterium tumefaciens* was added for re-suspension, shaken, and infected for 4 min. It was then decanted and the bacteria liquid was thoroughly absorbed with sterile filter paper, and then the cotyledons were transferred to pre-culture medium for dark culture for 2 days. Cotyledons were then cultured on 2 Z medium for c.15 days, and after callus formation, they were cultured on 0.2 Z medium for redifferentiation and screening, and subcultured once for c.15 days. When adventitious buds had grown to 2~3 cm, they were transferred to rooting medium to induce rooting. Transplant transgenic seedlings were planted in nutrient soil, and cultured in the greenhouse.

### 4.3. RT-qPCR Detection and Analysis

*SlWRKY3* transgenic line and control line (WT) seeds were evenly placed in a culture medium, covered with filter paper, and then transferred to a 28 °C incubator for germination. After germination, they were planted in plug trays, and transferred to the laboratory greening room. When the seedlings were c. 20 cm, *B. cinerea* was inoculated in vivo (i.e., sprayed with *B. cinerea* spore suspension), and the young leaves were taken and stored in liquid nitrogen at 0, 6, 12, 24, and 48 h after inoculation. RNA was extracted by Trizol reagent (Invitrogen, Waltham, MA, USA); cDNA was obtained by reverse transcription. Real-time quantitative reverse transcription polymerase chain reaction (RT-qPCR) was used to detect gene transcription levels, and Roche LightCycler 480 system was used to detect the relative gene expression at different time points after inoculation with *B. cinerea* [51]. The primer sequence design and experimental procedure for RT-qPCR are based on previous studies [52]. When designing RT-qPCR primers, we take into account that the amplification fragment is a specific segment of the gene, with the length of the amplified product ranging from 80 to 150 bp. The primers for RT-qPCR are listed in Supplementary Table S2. *Actin* gene (SGN-U580609) expression was used as an internal control. The reaction system, a total volume of 10 µL, was carefully assembled, consisting of a 5 µL SYBR Green master mix, 0.5 µL each of 10 µM of forward and reverse primers, and 4 µL of cDNA. The amplification protocol commenced with an initial denaturation at 95 °C for 60 s, succeeded by 45 cycles comprising denaturation at 95 °C for 10 s, annealing at 58 °C for 15 s, and extension at 72 °C for 20 s. The relative expression levels were quantified using the 2^−△△Ct^ method [51].

### 4.4. Yeast One-Hybrid Assays (Y1H)

The full-length *ERF118-like*, *SlWRKY3*, *JERF*, *B-box*, and *C3H4* were amplified with tomato cDNA as the template and inserted into *pGADT7* to obtain the prey vector (*ERF118-like*, *JERF*, *BPE-like*, *bHLH130-like*, *SlWRKY3-AD*). Fragments of the *TPK1b* promoters were amplified with tomato genomic DNA as the template and cloned into *pAbAi* to obtain a bait vector (*pAbAi-TPK1b*). The primer list of *pAbAi-TPK1b* and different truncated *pAbAi-TPK1b* and *SlWRKY3-AD* is provided in Supplementary Table S1. The yeast one-hybrid assays were performed using the Matchmaker Gold Yeast One-Hybrid System as previously described [53]. The yeast strain Y1H Gold was cultured in 2 × YPDA liquid medium at 28 °C, and shaken at 200 r·min^−1^ on a shaking table for 14 h. Bacterial fluid was collected twice by centrifuging at 12,000 r·min^−1^ for 30 s. An amount of 2 g·L^−1^ carrier DNA was prepared at 100 °C for 5 min, then denatured and placed on ice for later use. In the centrifuge tube of step 1, 50% PEG3350 240 μL, 1 mol·L^−1^ LiAc 34 μL, 2 g·L^−1^ carrier DNA 50 μL, and plasmid 10 μL were added and then the tube was soaked in water at 42 °C for 90 min. The sample was centrifuged at 12000 r·min^−1^ for 1 min, and 500 μL ddH_2_O was added into the centrifuge tube. A 100 μL bacterial solution was absorbed and evenly coated on SD/-Leu-Ura medium, and cultured at 30 °C for 3 d. Plates were examined after 3 d.

### 4.5. Identification of B. cinerea Resistance in Transgenic Plants

Transgenic tomato plants and WT were inoculated with *B. cinerea* as previously described [6], and then the third and fourth compound leaves from the top were inoculated with 10^5^ mL^−1^ suspension of *B. cinerea* spores. After 2~3 days, disease status was observed and photographed. Disease lesion size (mm^2^) was calculated using LA-S software (Hangzhou Wanshen Detection Technology Co., Ltd., China) and compared with WT. Then, plant resistance was analyzed.

### 4.6. Detection of Hydrogen Peroxide Content

Detached leaves of transgenic *SlWRKY3* and WT were inoculated with *B. cinerea*. Leaf samples were collected at 48 h and 72 h after inoculation, and stored in DAB (3,3’-diaminobenzidine) solution for staining [54]. The accumulation of reactive oxygen species was observed. Bottles were sealed and kept at 25 °C for 12 to 24 h under dark conditions. During this time, the dye solution was shaken several times to ensure full contact with the leaves. After incubation, the leaves were removed and bathed in 95% ethanol and heated in boiling water until the leaf chlorophyll had completely faded. The grayish brown spots formed by the reaction with DAB on leaves were observed and compared.

### 4.7. Statistical Analysis

GraphPad prism 8.0, SPSS 26.0 and Microsoft Excel were used for statistical analysis. All experiments were repeated at least three times. Statistically significant differences were determined by one-way ANOVA. Data are reported as mean ± SE. * indicates *p* < 0.05, and ** indicates *p* < 0.01. Different lowercase letters indicate significant differences.

## 5. Conclusions

Herein, our research showed that SlWRKY3 plays a pivotal role in the regulation of tomato resistance to *B. cinerea* by repressing *TPK1b* transcription, which in turn affects the SA and ROS pathways. This regulatory mechanism provides valuable insights into the complex interactions between plants and pathogens and can inform strategies for developing tomatoes with improved resistance to *B. cinerea*. Understanding these molecular mechanisms can guide future breeding efforts and genetic engineering approaches to enhance crop resistance to diseases.

## Figures and Tables

**Table 1:** *TPK1b* promoter yeast one-hybrid screens.

Bait Gene	SGN Accession Number	Name	Description
TPK1b	Solyc03g123500.2.1	JERF3	Pathogenesis-related transcriptional factor and ERF
	Solyc01g086870.2.1	bHLH130	Basic helix-loop-helix (bHLH) family transcription factor
	Solyc05g006650.2.1	BPE-like	Basic helix-loop-helix (bHLH) family transcription factor
	Solyc04g007180.1.1	ERF118	Ethylene-responsive transcription factor ERF118
	Solyc02g088340.2.1	WRKY3	WRKY family transcription factor, WRKY transcription factor 3

## Data Availability

Data are contained within the article and Supplementary Materials.

## Supplementary Materials

The following supporting information can be downloaded at: https://www.mdpi.com/article/10.3390/plants13121597/s1, Table S1. List of primers used in this study; Table S2. qRT-PCR primer sequences used to quantify the expression genes.
