# Supplementary material for: Tomato SlWRKY3 Negatively Regulates Botrytis cinerea Resistance via TPK1b
Source: Plants (Basel). 2024 Jun 8;13(12):1597. doi: 10.3390/plants13121597 (PMC11207927; doi:10.3390/plants13121597)
Supplement: Supplementary file 1 [file plants-13-01597-s001.zip › plants-2980474-supplementary.pdf]

Supplementary Data

Table S1. List of primers used in this study.

Primer name	Primer sequence (5’-3’)
TPK1b-Pabai-Fw	AGCTTGAATTCGAGCTCGGTACCAGTTACACTTTGTGGCTCAT
TPK1b-Pabai-Rv	ACATACAGAGCACATGCCTCGAGTAGAACAACAACAAACAAAA
SlWRKY3-OE-Fw	CATTTGGAGAGGACACGCTCGAGATGGGGGAAACAGGGGAAGCT
SlWRKY3-OE-Rv	TCTCATTAAAGCAGGACTCTAGATTAGCTGTATTTATTCCTTGT
SlWRKY3-Ri-Fw	GGGGACAAGTTTGTACAAAAAAGCAGGCTTTCCACCTCGCCCTTC ATTT
SlWRKY3-Ri-Rv	GGGGACCACTTTGTACAAGAAAGCTGGGTTCAAACCAGAGGGAAT AACGAAC
SlWRKY3-AD-Fw	ACGTACCAGATTACGCTCATATGATGGGGGAAACAGGGGAAGCT
SlWRKY3-AD-Rv	TACGATTCATCTGCAGCTCGAGCGCTGTATTTATTCCTTGTTCC
TPK1b-a-Pabai-Fw	AGCTTGAATTCGAGCTCGGTACCAGTTACACTTTGTGGCTCAT
TPK1b-a-Pabai-Rv	ACATACAGAGCACATGCCTCGAGTCACACCCAACCAACATTT
TPK1b-b-Pabai-Fw	AGCTTGAATTCGAGCTCGGTACCCTCTATCCCAACACCCCATC
TPK1b-b-Pabai-Rv	ACATACAGAGCACATGCCTCGAGAAAGGACCTTTCACTATT
TPK1b-c-Pabai-Fw	AGCTTGAATTCGAGCTCGGTACCAATAGTGAAAGGTCCTTT
TPK1b-c-Pabai-Rv	ACATACAGAGCACATGCCTCGAGCATTGTGCCCTTCCATTAC
TPK1b-D-Pabai-Fw	AGCTTGAATTCGAGCTCGGTACCTAAAATCTTTAGGGAAAT

Table S2. qRT-PCR primer sequences used to quantify the expression genes.

**Primer name**	**Primer sequence (5'-3')**
Q-actin-Fw	GTCCTCTTCCAGCCATCCAT
Q-actin-Rv	ACCACTGAGCACAATGTTACCG
Q-SlWRKY3-Fw	GGACAGTAATGAATAGCTCGGA
Q-SlWRKY3-Rv	TTGGATTCAGCTACTGTGACAT
Q-TPK1b-Fw	CAAGTAGTTTGTGTGTTCTGGG
Q-TPK1b-Rv	CAGATCTTGAATTTAGCCCTGC
Q-PR1-Fw	ACTTGGCATCCCGAGCACAA
Q-PR1-Rv	CTCGGACACCCACAATTGCA
Q-CAPX-Fw	GACTCTTGGAGCCCATTAGG
Q-CAPX-Rv	AGGGTGAAAGGGAACATCAG
